# Editorial: Community series in epigenetic regulation in cardiovascular diseases, volume III

**DOI:** 10.3389/fcvm.2024.1406370

**Published:** 2024-04-16

**Authors:** Zhihua Wang, Qing Robert Miao, Suowen Xu, Indulekha C. L. Pillai, Christoph D. Rau

**Affiliations:** ^1^ Shenzhen Key Laboratory of Cardiovascular Disease, Fuwai Hospital Chinese Academy of Medical Sciences, Shenzhen, China; ^2^ State Key Laboratory of Cardiovascular Disease, National Center for Cardiovascular Disease, Fuwai Hospital, Chinese Academy of Medical Sciences and Peking Union Medical College, Beijing, China; ^3^ Grossman Long Island School of Medicine, New York University, Mineola, NY, United States; ^4^ Institute of Endocrine and Metabolic Diseases, The First Affiliated Hospital of USTC, University of Science and Technology of China, Hefei, China; ^5^ Stem Cells and Regenerative Biology Laboratory, School of Biotechnology, Amrita Vishwa Vidyapeetham, Kollam, India; ^6^ Computational Medicine Program and Department of Genetics, The University of North Carolina at Chapel Hill, Chapel Hill, NC, United States

**Keywords:** cardiovascular diseases, epigenetics, chromatin remodelling, histone modifications, DNA methylation

Editorial on the Research Topic Community series in epigenetic regulation in cardiovascular diseases, volume III

## Main

Epigenetic mechanisms contribute to the gene expression abnormality underlying multiple cardiovascular diseases (CVDs). Unveiling novel epigenetic players and their interplays governing gene reprogramming during the pathogenesis of CVDs remains a cutting-edge topic in the field. In the third volume of our research topic series, we collected four high-quality papers, including one research article and three reviews, discussing the roles of transcription factors, epigenetic modifiers, non-coding RNAs, and RNA modifications in different types of CVDs. These novel findings and insights in this collection represent on-time instructions to renew our understanding of cardiovascular epigenetics under pathophysiological conditions.

The increasing global prevalence of obesity and diabetes leads to an epidemic rise of diabetic cardiomyopathy, which has become a prominent medical challenge, particularly in the context of global population ageing. The molecular links between metabolic disorders and cardiovascular complications have not yet been fully resolved. Transcription factor Tumor Protein p53-inducible Nuclear Protein 2 (Trp53inp2) has previously been identified as a candidate gene linking glucose utilization and cardiomyocyte hypertrophy through an unbiased systems genetics study in mice. However, direct evidence still needs to be included. Here, Liu et al. developed an inducible Trp53inp2 knockout mouse line and investigated the impact of Trp53inp2 inactivation on the pathogenesis of heart failure under mechanical and/or metabolic stresses. Surprisingly, Trp53inp2 inactivation in the heart led to the accelerated onset of heart failure with reduced ejection fraction (HFrEF) in response to pressure overload, but it ameliorated cardiac dysfunction induced by combined stresses of high-fat diet and moderate pressure overload (so-called Cardiometabolic Disorder Model) due to the differential regulation of glucose metabolism genes. This study provides a biological basis to bridge metabolic stresses and cardiovascular outcomes.

Obesity is a major risk factor for heart failure with preserved ejection fraction (HFpEF), which attracts more and more attention in current cardiovascular medicine. Despite decades of research, the treatment option for HFpEF still needs to be improved due to our incomplete understanding of the underlying molecular mechanisms. In a review written by Jalink et al. the authors summarized the recent advances in the role of non-coding RNAs, including miRNAs, lncRNAs, and circRNAs, in the pathophysiology of HFpEF by interrogating clinical evidence and dissecting the molecular mechanisms of the ncRNAs, the authors proposed the potential of ncRNAs as biomarkers and potential novel therapeutic targets for future HFpEF treatments.

Although RNA modifications have long been studied on housekeeping RNAs, like tRNAs and rRNAs, we only recently recognized the important physiological function of the modifications, particularly methylation, on mRNAs. N6-methyladenosine (m^6^A) methylation is the most common epigenetic modification on mRNAs that has been extensively investigated in diverse disease models. Zhang et al. provide a comprehensive review, summarizing recent advances in the role of mRNA m^6^A modification in CVDs. The authors digested the regulations and actions by m^6^A writers, erasers, and readers in different biological aspects, including calcium homeostasis, endothelial function, cell death, autophagy, endoplasmic reticulum stress, macrophage response and inflammation, in CVDs, especially in heart failure and ischemic heart disease. Current evidence suggests that targeting m^6^A modifiers could hold translational therapeutic value in treating CVDs.

Sexual differences in heart disease are a significant area of interest within the realm of personalized medicine. Despite clear variations in how heart conditions manifest and progress between genders, a historical lack of attention to gender-specific factors has meant that patient treatment has only sometimes been tailored accordingly. This oversight can be attributed to intensive emphasis on male participants in research studies and a failure to categorize findings by sex. Epigenetics has emerged as a key player in shedding light on the differences between males and females in terms of both heart health and disease. A review by Bridges et al. thoroughly examines the biological variances between men and women in the development of various cardiac complications, particularly focusing on uncovering the genetic and epigenetic mechanisms at play. The authors digested current findings regarding unique sex-chromosome compositions, the emergence of genetic variations that exhibit gender bias, and variations in hormonal profiles between the sexes, with organized perspectives from DNA methylation, histone marks, and chromatin structure. This review raises critical concerns about the long-standing neglect of gender differences in cardiovascular disease research. Elucidating how sex-specific genetic, hormonal, and physiological factors contribute to the development and progression of cardiovascular diseases would not only advance our overall understanding of disease mechanisms but also open up new avenues for personalized medicine approaches.

The papers we have gathered in conjunction with the two preceding volumes of this research topic comprehensively address the various aspects we initially aimed to explore when we embarked on this endeavor three years ago. They collectively provide a current overview of the cutting-edge research in the field of epigenetics related to diverse cardiovascular diseases. These studies have yielded valuable findings that shed new light on the understanding of cardiovascular pathologies and undoubtedly hold promise for advancing the development of innovative therapeutic approaches to tackle these specific cardiovascular conditions.

